# Mitigation of Salt Stress in Rice by the Halotolerant Plant Growth-Promoting Bacterium *Enterobacter asburiae* D2

**DOI:** 10.3390/jox14010021

**Published:** 2024-03-01

**Authors:** Zican Ning, Kexin Lin, Mengya Gao, Xiao Han, Qingjie Guan, Xiang Ji, Shuyu Yu, Lei Lu

**Affiliations:** 1 College of Life Sciences, Northeast Forestry University, Harbin 150040, China; zicanning@163.com (Z.N.); kx_lin@126.com (K.L.); mengyagao@yeah.net (M.G.); xiaohan093@163.com (X.H.); qingjieguan@126.com (Q.G.); 2 Key Laboratory of Saline-Alkali Vegetation Ecology Restoration, Ministry of Education, Northeast Forestry University, Harbin 150040, China; 3 College of Water Conservancy and Civil Engineering, Inner Mongolia Agricultural University, Hohhot 010018, China; jixiang@imau.edu.cn; 4 Hetao College, Bayannur 015000, China

**Keywords:** plant growth-promoting rhizobacteria, rice, *Enterobacter asburiae*, genome analysis, salt stress

## Abstract

Salinity is a major abiotic stress that seriously affects crop growth worldwide. In this work, we aimed to isolate potential halotolerant plant growth-promoting rhizobacteria (PGPR) to mitigate the adverse impacts of salt stress in rice. An isolate, D2, with multiple plant growth-promoting (PGP) characteristics was identified as *Enterobacter asburiae* D2. Strain D2 could produce indole-3-acetic acid and siderophore. It also exhibited phosphate solubilization and 1-aminocyclopropane-1-carboxylic deaminase activity. Genome analysis further provided insights into the molecular mechanism of its PGP abilities. Strain D2 inoculation efficiently stimulated rice growth under both normal and saline conditions. Compared with the non-inoculated plants, a significant increase in plant height (18.1–34.7%), root length (25.9–57.1%), root dry weight (57.1–150%), and shoot dry weight (17.3–50.4%) was recorded in inoculated rice seedlings. Meanwhile, rice seedlings inoculated with strain D2 showed improvement in chlorophyll and proline content, while the oxidant damage was reduced in these plants in comparison with the control group. Moreover, the K^+^/Na^+^ ratio of the inoculated rice seedlings exposed to NaCl and Na_2_CO_3_ was higher than that of the uninoculated groups. These results imply that *Enterobacter asburiae* D2 is a potential PGPR that can be used for alleviation of salt stress in rice.

## 1. Introduction

Rice (*Oryza sativa* L.) is a premier staple food for more than half of the world’s population. It is essential to increase rice production to support the huge food demand of a steadily growing global population [1]. However, the productivity of rice is threatened by a number of abiotic stresses due to anthropogenic practices and climate change. In particular, salt stress is considered a predominant abiotic threat to rice production around the world, as more than 20% of the global agricultural soil is affected by salinity [2]. Rice is inherently sensitive to salinity during its entire growth span, including seed germination, seedling growth, and reproductive development. Excessive salt in the soil causes strong detrimental effects on rice, such as lowering nutrient availability, disrupting ion balance, inhibiting photosynthesis, and causing oxidative stress [1,2]. Consequently, salt stress results in great damage to rice growth, development, and yield. Various strategies have been explored to ameliorate rice resistance to salt stress, including the breeding of salt-tolerant varieties, application of chemical amendments, and inoculation of beneficial microorganisms [3,4,5]. Among these approaches, using plant growth-promoting rhizobacteria (PGPR) has gained substantial attention as an efficient and natural solution to tackle salt stress in plants and improve crop productivity [6].

In nature, PGPR inhabit the rhizosphere region and usually develop beneficial interactions with plants, which plays an important role in plant growth and response to abiotic stresses [6,7]. PGPR stimulate plant growth under stressful environments via different mechanisms. They can facilitate plant nutrient uptake through nitrogen fixation, mineral solubilization, and siderophore production. Some PGPR synthesize phytohormones like indole-3-acetic acid (IAA) to promote plant growth and development [6,7]. Also, PGPR possessing 1-aminocyclopropane-1-carboxylic (ACC) deaminase activity can degrade the plant ethylene precursor ACC, and thereby reduce the stress-induced ethylene content in plants [6,7,8]. Under saline environments, PGPR can alleviate the adverse effects of salt stress in plants by maintaining ionic homeostasis, enhancing osmolyte accumulation, and reducing oxidative stress [7,8].

To improve plant growth in high-salt environments, PGPR need to be halotolerant to cope with osmotic stress. Recently, various halotolerant PGPR such as *Enterobacter*, *Bacillus*, *Pseudomonas*, *Klebsiella*, and *Acinetobacter* have been isolated from different environments, which exhibited a strong ability to promote rice growth in saline conditions [5,9,10,11]. For example, the inoculation of the salt-tolerant *Bacillus pumilus* strain JPVS11 significantly enhanced the plant height, root length, plant biomass, and chlorophyll content at NaCl concentrations from 50 to 300 mM [10]. Khumairah et al. isolated fifteen halotolerant PGPR from the rhizosphere of rice, mangroves, and wild grass. All the isolates could produce varying amounts of IAA and promote rice growth under saline environments [5]. To date, the application of halotolerant PGPR has mainly focused on their ability to improve plant growth at high NaCl concentrations [8].

Salt-affected soil is generally formed by the accumulation of neutral and/or alkaline salts. In some areas like Northeast China, the main salt components in salinized soil are NaHCO_3_ and Na_2_CO_3_, which make the soil pH alkaline (mostly higher than pH 8.5) [12]. Except for the osmotic and ionic stresses, alkaline salts also result in high pH stress in plants, which not only directly damages plants but also seriously interferes with the absorption of mineral nutrition [12]. Therefore, the stress induced by alkaline salts causes higher inhibition of plant growth than the neutral salts. The beneficial effects of halotolerant PGPR on plant growth require further exploration in alkaline conditions. In this work, we aim to isolate halotolerant PGPR and evaluate their ability to promote rice growth under both neutral and alkaline salt stresses. We identified and characterized the plant growth-promoting (PGP) traits of the selected isolate. Furthermore, we investigated the effects of strain inoculation on the growth performance and physiological responses of rice under salt stress.

## 2. Materials and Methods

### 2.1. Isolation of Halotolerant Rhizobacteria from Rice

The rhizosphere soil was collected from rice cultivated in Daqing, Heilongjiang Province, China (124°38′59″ E, 45°38′48″ N). The soil samples were stored in aseptic bags on ice and transferred to the laboratory for subsequent processing. Isolation of halotolerant bacteria was conducted by serially diluting the soil samples to 10^−6^ in sterilized NaCl solution (0.85%). Aliquots (150 μL) from each dilution were spread on tryptic soy agar (TSA) supplemented with 3% NaCl and the pH was adjusted to 8.5. The plates were then incubated at 30 °C for 24–48 h. Single colonies with distinct morphologies were selected, purified, and then maintained in 20% glycerol at −70 °C. The PGP attributes of the isolated strains, including phosphate solubilization, siderophore production, and ACC deaminase activity, were preliminarily evaluated on agar plates [13,14]. Briefly, the phosphate solubilization ability was estimated on Pikovskaya agar medium containing tricalcium phosphate. The clear halo zones around the colonies were measured after 5 days of incubation at 30 °C [13,14]. Siderophore production was checked by the formation of orange halos around bacterial colonies on Chrome Azurol S (CAS) agar plates after incubation at 30 °C for 24 h [13,14]. The production of ACC deaminase was detected by the growth of bacterial strains on Dworkin–Foster (DF) agar medium with ACC as the nitrogen source at 30 °C for 48 h [14]. The bacterial strain with multiple PGP properties was used for further analysis.

### 2.2. Morphological and Biochemical Characteristics

The morphological characteristics of the isolate were observed with an optical microscope using conventional Gram-staining procedures. The biochemical assays such as oxidase test, nitrate reduction, and citrate utilization were conducted using the Biochemical Identification Tube System according to the instructions of the manufacturer (Hopebio, Qingdao, China). The halotolerance of the isolated strain was evaluated by observing its growth in the Luria-Bertani (LB) medium containing different concentrations of NaCl (0.5–1.5 M). The effects of pH on bacterial growth were investigated using the LB medium with pH ranging from 7.0 to 11.0. The isolate was grown overnight at 30 °C, and the growth was recorded at 600 nm by a spectrophotometer. The temperature range for growth was determined by incubation of the isolate in the LB medium at 20–40 °C.

### 2.3. Identification of the Selected Isolate

The genomic DNA of the selected strain was extracted with the EasyPure Genomic DNA Kit (TransGen Biotech, Beijing, China). The 16S rRNA gene was amplified with the genomic DNA as a template using the universal primers fD1 (5′-AGAGTTTGATCCTGGCTCAG-3′) and rP1 (5′-ACGGTTACCTTGTTACGACTT-3′) [15]. PCR amplification was performed with an initial denaturation at 94 °C for 4 min, 30 cycles of 1 min at 94 °C, 1 min at 55 °C, 2 min at 72 °C, and a final extension for 10 min at 72 °C. The PCR products were sent to Comate Bioscience (Changchun, China) for sequencing. The obtained sequence was analyzed by sequence similarity search using the EzBioCloud database [16]. The phylogenetic tree was then constructed based on the neighbor-joining method using MEGA software (version X) [17].

### 2.4. Genome Sequencing and Analysis

The genome of the isolated strain was sequenced using a paired-end strategy (2 × 150 bp) on an Illumina Hiseq platform by the Majorbio Bio-pharm Technology Co., Ltd. (Shanghai, China). The data were analyzed on the online platform of Majorbio Cloud Platform [18]. The raw data were quality controlled to obtain high-quality clean data. A coverage of 227× was achieved during the sequencing, and the filtered reads were assembled using SOAPdenovo (v2.04). The coding sequences (CDS) of the bacterial genome were predicted with Prodigal (v2.6.3). Prediction of tRNA and rRNA was performed using tRNAscan-SE (v2.0.12) and Barmap (v0.9), respectively. Annotation of the predicted genes was performed using different databases, including the Non-redundant Protein Database (NR), Swiss-Prot, Pfam, Gene Ontology (GO), Clusters of Orthologous Genes (COG), and Kyoto Encyclopedia of Genes and Genomes (KEGG). The circular genome map was generated using CGView (v2). To further identify strain D2, average nucleotide identity (ANI) was determined to evaluate genome similarities using ANI Calculator [16]. The genome sequences of closely related *Enterobacter* strains were obtained from the EzBioCloud database [16].

### 2.5. Evaluation of Plant Growth-Promoting Attributes

The properties including IAA and siderophore production, phosphate solubilization, and ACC deaminase activity were determined to quantify the PGP ability of the isolated strain. IAA production was carried out in LB broth added with or without (control) 500 mg/L of L-tryptophan. The IAA content in the culture supernatant was determined at 535 nm using the Salkowski reagent [19]. Siderophore production was performed by incubation the strain in a modified King’s B (MKB) medium, and then the produced siderophore was detected following the Chrome Azurol S (CAS) shuttle assay [20]. The phosphate solubilization ability was investigated in the National Botanical Research Institute’s phosphate growth medium (NBRIP) [21]. The inorganic phosphate in the cell-free supernatant was quantified by the molybdenum blue method [22]. ACC deaminase activity was assayed according to the method described by Penrose and Glick [23]. The protein content in the sample was determined by the Bradford Protein Assay Kit (Tiangen, Beijing, China). The ACC deaminase activity was defined as the amount of α-keto-butyrate produced per mg of protein per h.

### 2.6. Effect of Strain Inoculation on Rice Growth under Salt Stress

#### 2.6.1. Plant Inoculation and Salt Treatment

The rice seeds of cultivar Changbai 9 were surface-sterilized with 70% ethanol for 2 min and 3% NaClO for 5 min, followed by washing five times with autoclaved distilled water. The sterile rice seeds were germinated in Petri dishes containing two layers of filter paper that were moistened with sterile distilled water. Five-day-old seedlings with similar growth status were transplanted in plastic pots (five seedlings in each pot) filled with an autoclaved mixture of soil and vermiculite (3:1). The pots were placed in a plant growth room at 30 °C and a 16/8 h light/dark condition. Plant inoculation was carried out according to Chatterjee et al. [24] with some modifications. The bacterium was grown in LB broth overnight at 30 °C, 180 rpm. The cell pellets were collected by centrifugation, and diluted with sterile MgSO_4_ (30 mM) to an OD_600_ of 1.0. The bacterial suspension (20 mL) was applied to the roots of the seedlings at 1 day and 7 days after transplantation. Uninoculated seedlings were used as control and were irrigated with the same amount of 30 mM MgSO_4_. Salt stress was conducted after the second inoculation by watering 30 mL of 150 mM NaCl or 40 mM Na_2_CO_3_ solution at intervals of 2 days. A group without salt treatment was also set by normally irrigated with distilled water. Each treatment contained five pots. After 15 days of salt treatment, the plants were collected and the growth parameters like plant height and biomass were recorded. To measure the dry weight of seedlings, the shoot and root samples were dried in an oven at 70 °C for 48 h and then the weight of the samples was recorded.

#### 2.6.2. Biochemical Analysis of Plants

The chlorophyll content was estimated by homogenizing fresh leaves with 80% acetone in a pestle and mortar. The absorbance of the supernatant was measured at 645 and 663 nm to calculate the chlorophyll contents [25]. Proline content was evaluated by crushing fresh leaves in 3% sulfosalicylic acid. Then, the supernatant obtained by centrifugation was mixed with glacial acetic acid and ninhydrin (1:1, *v*/*v*). The mixture was incubated in a boiling water bath for 1 h and was extracted with toluene after cooling. The absorbance of the extract was recorded at 520 nm, and the proline concentration was calculated from a standard curve of pure proline [26]. Malondialdehyde (MDA) content in the leaves was analyzed by the thiobarbutaric acid (TBA) method using the MDA assay kit provided by Jiancheng Bioengineering Institute (Nanjing, China).

The crude protein of the leaves was extracted by grounding the fresh samples in pre-chilled phosphate buffer (pH 7.4). The supernatant was obtained by centrifugation at 10,000 rpm for 10 min and was used for assay of protein content and enzymatic activities. Antioxidant enzymes activities of catalase (CAT), superoxide dismutase (SOD), and peroxidase (POD) in fresh leaves were measured by the related assay kits following the manufacturer’s structures (Jiancheng Bioengineering Institute, Nanjing, China).

To measure the changes in ion contents (Na^+^ and K^+^) in rice, the leaves were oven-dried at 80 °C to constant weight. The dry samples were ground and digested in nitric acid and hydrogen peroxide [27]. Then, the ion contents were determined by an atomic absorption spectrophotometer (PinAAcle 900T, PerkinElmer, Waltham, MA, USA).

### 2.7. Statistical Analysis

All the assays were conducted in triplicate. Statistical analysis was conducted using the SPSS 21.0 package. Data was subjected to analysis of variance (ANOVA) followed by Duncan’s test at *p* < 0.05.

## 3. Results

### 3.1. Isolation, Characterization and Identification of Strain D2

**Figure 1:**
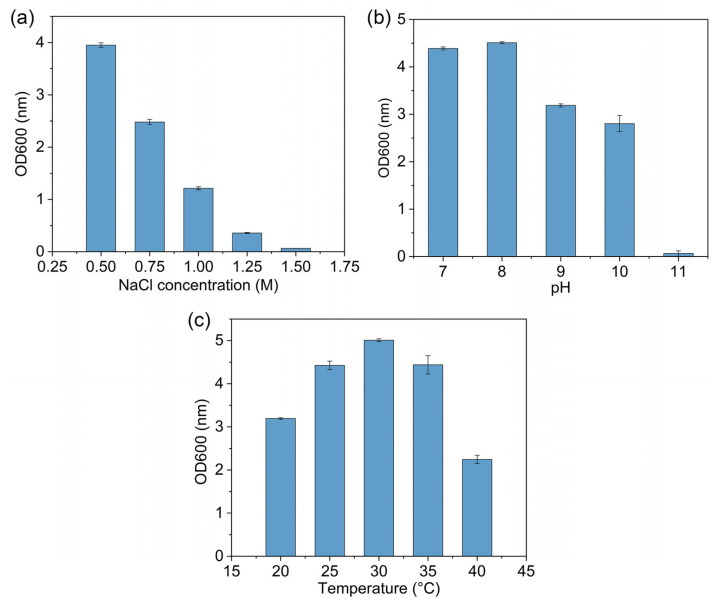
Effect of NaCl concentration (**a**), pH (**b**), and temperature (**c**) on the growth of strain D2 in the LB medium.

**Figure 2:**
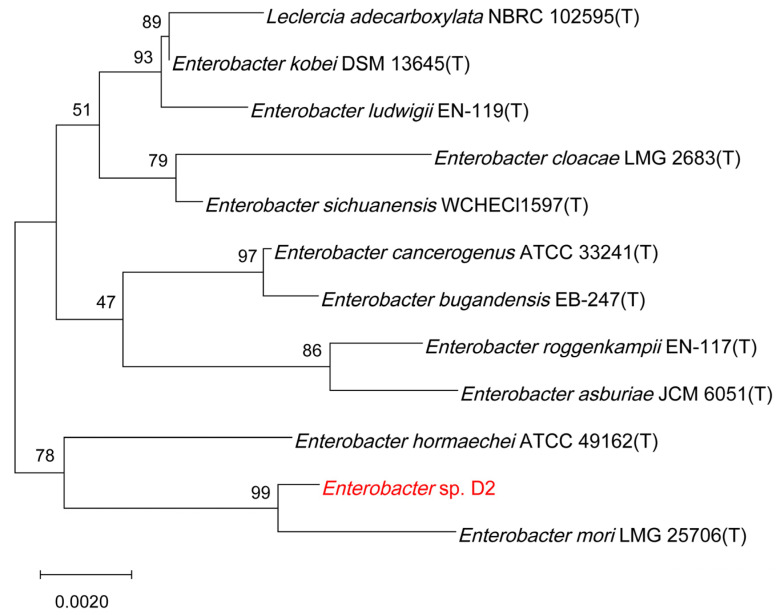
Phylogenetic tree of strain D2 based on 16S rRNA gene sequence using neighbor-joining method.

Six halotolerant bacteria with different morphological characteristics were isolated from the rhizosphere of rice. Among these isolates, strain D2 was found to possess multiple PGP traits and was further analyzed for strain characteristics and identification (Table S1). Strain D2 was a Gram-negative and rod-shaped bacterium. After growing on the TSA plate for 48 h at 30 °C, the isolate formed creamy-white, convex, round, and smooth colonies with diameters of 1–2 mm. The strain showed positive results for lysine decarboxylase, β-galactosidase, arginine dihydrolase, nitrate reduction, Voges-Proskauer test, gelatin hydrolysis, and malonate and citrate utilization. It could produce acids from glucose, fructose, mannitol, sorbitol, and melibiose (Table 1). Strain D2 could grow at a NaCl concentration of 1.25 M (Figure 1a). It also exhibited good tolerance to alkaline environments, with the ability to survive with a pH of up to 10 (Figure 1b). The isolate D2 showed optimal growth in the temperature range of 25–35 °C (Figure 1c). Identification of the strain was conducted by 16S rRNA gene sequence analysis, which has been submitted to the GenBank database with the accession number MN540931. Sequence analysis revealed that strain D2 had the highest similarity with *Enterobacter mori* LMG 25706(T) (99.52%). High similarities of 16S rRNA gene sequences were also found between strain D2 and other species from *Enterobacter* genus. The phylogenetic tree reflected that strain D2 had high homology with *Enterobacter mori* LMG 25706(T) (Figure 2). Therefore, strain D2 was classified as *Enterobacter* sp.

### 3.2. Genome Analysis of Strain D2

#### 3.2.1. General Characteristics of the D2 Genome

**Figure 3:**
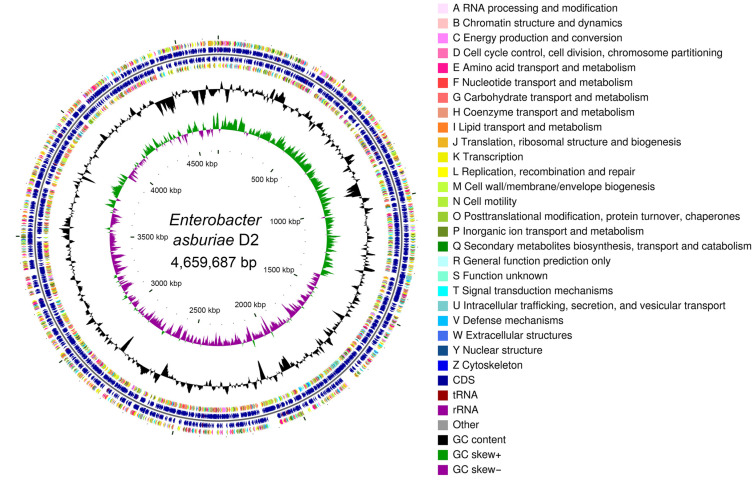
Genome map of *Enterobacter asburiae* D2. From the outside to the inside, the first and fourth circles are functional COG classifications on the positive and negative chains, respectively; the second and third circles are the coding sequences (CDSs) on the positive and negative chains, respectively; the fifth circle is the GC content; the sixth circle is the GC-skew value, and the innermost is the genome size indicator.

The genome of strain D2 consisted of 4,659,687 bp with an average G + C content of 55.73% (Figure 3). A total of 4327 protein-coding sequences, 74 tRNA genes, and 9 rRNA genes were predicted (Table S2). The whole-genome sequence was deposited to GenBank with the accession number of JAXQPP000000000. About 3779 functional genes were annotated by the COG database, which accounted for 87.34% of all genes and could be classified into 24 types (Figure 3). Metabolism categories such as amino acid, carbohydrate, and inorganic ion transport and metabolism possessed the highest number of genes (1844) (Figure S1). The number of genes annotated by the GO database was 3160, accounting for 73.03% of the total genes. These annotated genes were classified into three GO categories, including biological process (1871 genes), cellular component (1718 genes), and molecular function (2607 genes) (Figure S2). A total of 3082 genes of strain D2 were functionally annotated in the KEGG database, including 42 pathways of cellular processes, environmental information processing, genetic information processing, human diseases, metabolism, and organismal systems (Figure S3). Among the six categories of KEGG pathways, metabolism contained the largest number of genes (2300), followed by environmental information processing (488). A further taxonomical analysis of strain D2 was performed using average nucleotide identity (ANI) calculator to assess genome similarities [16]. The ANI values of strain D2 and other closely related *Enterobacter* species were determined (Table S3). According to a species boundary of 95% for ANI value [16], strain D2 was finally identified as *Enterobacter asburiae*.

#### 3.2.2. Genes Involved in Plant Growth Promotion and Stress Response

Analysis of the genome of strain D2 revealed the presence of some functional genes related to the promotion of plant growth (Table S4). Tryptophan is a precursor of bacterial IAA synthesis. A set of tryptophan biosynthesis genes were identified in the genome of this bacterium, including *trpA*, *trpB*, *trpCF*, *trpE*, *trpGD*, and *trpS*. In addition, a key gene involved in the IAA biosynthesis (*ipdC*) was found in the genome sequence of strain D2, which converts indole-3-pyruvate to indole-3-acetaldehyde. Besides the phytohormone IAA, the genes related to cytokinin synthesis (*miaA/B/E*) were detected in the genome of strain D2 (Table S4). The ACC deaminase gene (*acdS*) was not observed in strain D2. However, genome analysis revealed a gene (*dcyD*) encoding D-cysteine desulfhydrase, which is a homolog of ACC deaminase [28]. The strain carries genes related to inorganic phosphate transport and solubilization, such as *pstA/B/C/S* and *gcd*. It also has genes associated with organic phosphorus metabolism, including the *phn* genes, *phoA*, and *aphA* (Table S4). The nitrogen fixation-related genes (*nifJ*, *iscA/R/S/U*, and *sufA/B/C/D/E/S*) were found in the genome of D2, indicating the nitrogen fixation ability of this bacterium (Table S4). The gene cluster *entA/B/C/D/E/F/H/S* is responsible for the synthesis and transport of siderophore enterobactin. The presence of the *iucA/B/C/D* gene cluster leads to the biosynthesis of siderophore aerobactin. In addition, the *fepA/B/C/D/G* gene cluster acts as an iron-siderophore transport system. Several gene clusters involved in iron uptake and transport were also identified, including *fhuB/C/D*, *afuA/B/C*, and *efeB/O/U* (Table S4). The genome of strain D2 harbors various types of osmolytes synthesis and transport genes, such as glycine–betaine (*betA/B/T*), proline (*proA/B/C/P/S/V/W/X/Y*), and trehalose (*treB/S/Y/Z* and *otsA/B*). Furthermore, we identified several genes associated with bacterial exopolysaccharides (EPS) production, including cellulose (*bcsA/B/C/Z*), colanic acid (*wcaA/B/C/D/E/F/I/J/K/L/M*), and poly-β(1,6)-N-acetyl-D-glucosamine (*pgaA/B/C/D*) (Table S4).

### 3.3. Evaluation of the PGP Properties of Strain D2

The PGP traits of strain D2 were further validated in quantitative tests. Strain D2 could utilize L-tryptophan to produce IAA, with a production yield of 20.8 ± 1.8 μg/mL after 48 h of incubation. It showed a high capacity for solubilizing inorganic phosphate. About 548.8 ± 6.6 mg/L phosphorus was released from insoluble tricalcium phosphate at 72 h. Meanwhile, the pH of the culture supernatant dropped from 7.0 to 4.3 during the phosphate solubilization process. Despite strain D2 could grow on Dworkin–Foster (DF) agar plates with ACC as the sole nitrogen source, its ACC deaminase activity was rather low. Only 24.8 ± 4.9 nmol α-KB mg^−1^ h^−1^ of activity was detected after incubation in DF medium for 24 h. The siderophore production of strain D2 was quantified as 38.0 ± 2.5% after 48 h.

### 3.4. Effect of Strain D2 Inoculation on the Growth of Rice under Salt Stress

#### 3.4.1. Growth Parameters

**Figure 4:**
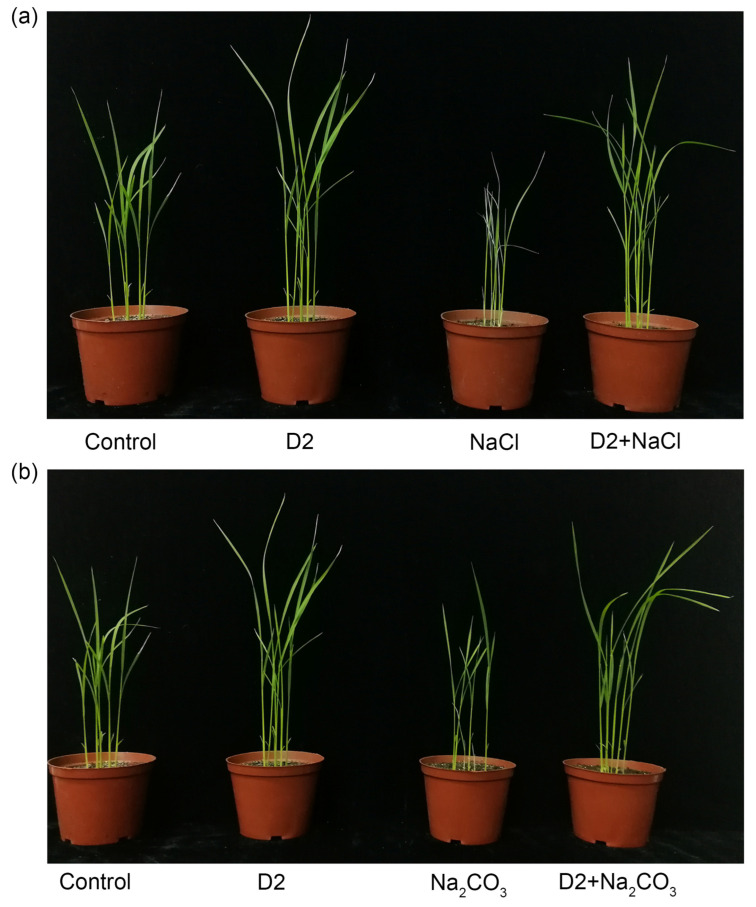
Effect of strain D2 inoculation on rice growth in the presence and absence of salt. (**a**) Salt treatment with 150 mM NaCl; (**b**) salt treatment with 40 mM Na_2_CO_3_.

Pot experiments were conducted to verify the PGP effects of strain D2 on rice growth under normal and saline conditions. Control experiments were performed without strain inoculation. Salt stress greatly inhibited the rice seedling growth in the absence of strain D2 (Figure 4). In the control group treated with 150 mM NaCl, the decrease in growth parameters of rice seedlings ranged between 23.2% and 60.7%. Similarly, the presence of 40 mM Na_2_CO_3_ caused a 10.7–42.9% decrease in the length and biomass of rice seedlings (Table 2). Compared with the control, bacterial inoculation significantly promoted the growth of rice seedlings under both salt and non-salt conditions (*p* < 0.05) (Figure 4). In particular, strain D2 showed a remarkable ability to enhance root growth regardless of salt treatment (Table 2). D2 inoculation increased the root length and dry weight by 25.9–57.1% and 57.1–150% in comparison with the control, respectively. The length and dry weight of the shoot were less affected by bacterial inoculation than those of the root, which were improved by 18.1–34.7% and 17.3–50.4%, respectively (Table 2). Strain D2 inoculation obviously alleviated the adverse effects induced by both the neutral and alkaline salt. For rice seedlings treated by strain inoculation and salt stress, most of the growth parameters were comparable to or even higher than those of the control plants grown under non-salt conditions (Table 2).

#### 3.4.2. Contents of Chlorophyll, Total Soluble Protein, Proline, and MDA

**Figure 5:**
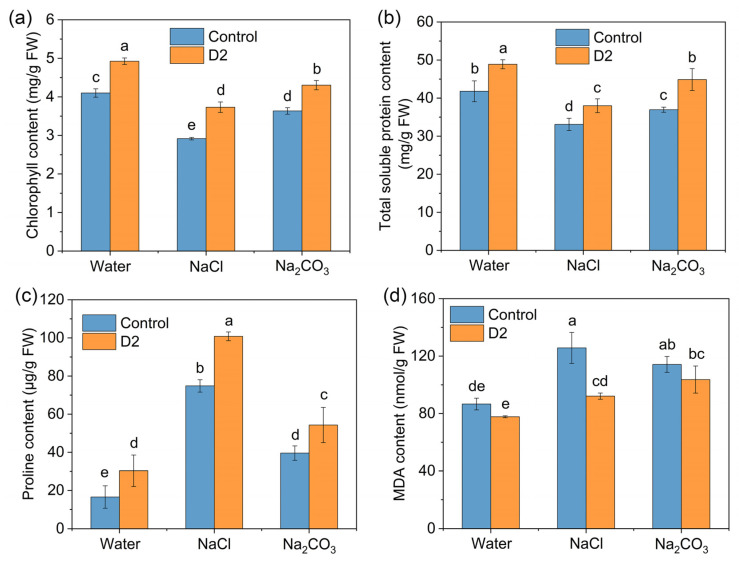
Quantification of the contents of (**a**) chlorophyll, (**b**) total soluble protein, (**c**) proline, and (**d**) MDA in rice with and without strain D2 inoculation. Different letters show statistically significant differences (*p* < 0.05).

To explore the response of rice to salt stress inoculated with D2, we examined the levels of some biochemical parameters including the contents of chlorophyll, total soluble protein, proline, and MDA, the activities of antioxidant enzymes, and the concentrations of Na^+^ and K^+^. Rice seedlings treated with NaCl and Na_2_CO_3_ resulted in a decrease of the chlorophyll content in leaves by 29% and 11.5%, respectively (Figure 5a). Nevertheless, D2-treated plants exhibited effective restoration of chlorophyll and significantly enhanced the chlorophyll content in all groups (*p* < 0.05). The total chlorophyll contents in the D2-inoculated group were 18.5–28.2% higher than those of the control group (Figure 5a). Similarly, salt stress induced a reduction in the content of total soluble protein. Under control conditions, the uninoculated rice seedlings had an average total soluble protein content of 41.8 mg/g. The soluble protein content of seedlings treated with NaCl and Na_2_CO_3_ decreased to 31.3 mg/g and 36.3 mg/g, respectively (Figure 5b). The application of D2 enhanced the soluble protein content of seedlings by 17%, 14.8%, and 21.4% for the respective control groups of water, NaCl, and Na_2_CO_3_.

Upon salt treatment, a significant accumulation of proline was observed in the rice leaves. The highest content of proline in the control group was found in the NaCl-treated seedlings, which was about 4.5 times higher than that of the seedlings irrigated with water (Figure 5c). Inoculation with strain D2 could significantly increase the proline content at all treatments (*p* < 0.05). The cotreatment with D2 and NaCl resulted in the highest accumulation of proline, achieving a content of 100.8 μg/g. Likewise, a significant increase in the MDA content of rice leaves was found under salt stress conditions. In the control group, the highest MDA content in rice was observed in NaCl treatment (125.7 nmol/g), followed by Na_2_CO_3_ treatment (114.2 nmol/g) and water treatment (86.6 nmol/g) (Figure 5d). Inoculation with D2 led to a reduction in MDA production in rice seedlings. The most prominent effect was also observed in the NaCl-treated group. Inoculation of D2 resulted in a 26.7% decrease in the MDA content (Figure 5d).

#### 3.4.3. Antioxidant Enzyme Activities

**Figure 6:**
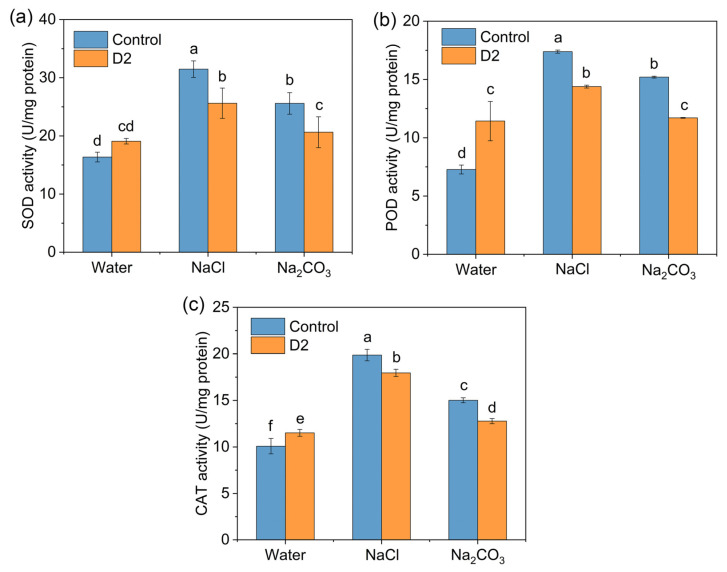
Changes in antioxidant enzyme activities in rice with and without strain D2 inoculation. (**a**) Superoxide dismutase (SOD); (**b**) peroxidase (POD); (**c**) catalase (CAT). Different letters show statistically significant differences (*p* < 0.05).

The activities of different antioxidant enzymes (SOD, POD, and CAT) were measured in rice plants grown under normal and saline environments. In the absence of D2, rice plants exposed to both NaCl and Na_2_CO_3_ stress caused a significantly higher activity of antioxidant enzymes when compared with the water group (Figure 6). An increase of 92.4% in SOD activity, 139.2% in POD activity, and 97.2% in CAT activity was observed in the NaCl treatment group. The Na_2_CO_3_ treatment enhanced the activities of SOD, POD, and CAT by 56.4%, 109.1%, and 48.9%, respectively (Figure 6). Bacterial inoculation showed a drop in the activities of these reactive oxygen species (ROS) scavenging enzymes by 9.7–23% in comparison with the control group of salt treatment, suggesting an alleviation of oxidative stress in the rice seedlings (Figure 6).

#### 3.4.4. Na^+^ and K^+^ Concentrations

**Figure 7:**
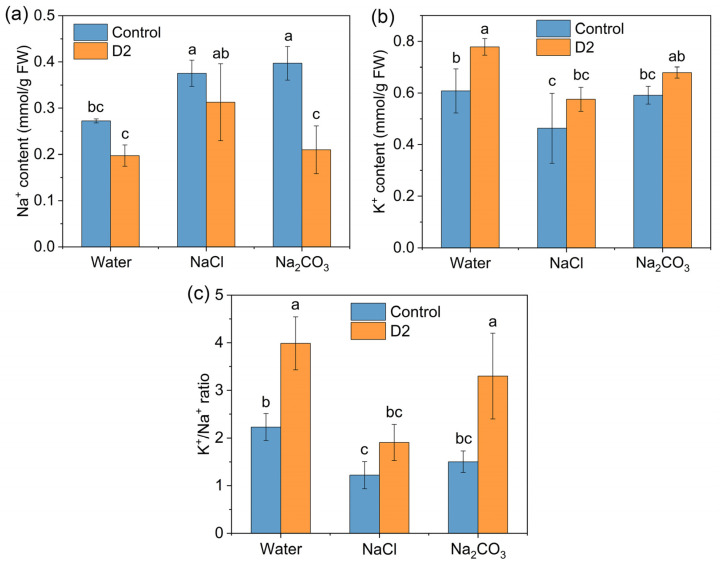
Effect of strain D2 inoculation on the ionic contents in rice. (**a**) Na^+^ concentration; (**b**) K^+^ concentration; (**c**) K^+^/Na^+^ ratio. Different letters show statistically significant differences (*p* < 0.05).

Changes in the Na^+^ and K^+^ concentrations were determined to explore the effect of bacterial inoculation on the ion uptake of rice seedlings under salt stress. An enhanced Na^+^ uptake was observed for rice exposed to both NaCl and Na_2_CO_3_, which was about 1.4 and 1.5 times higher than that of rice grown at normal conditions, respectively (Figure 7a). In contrast, salt stress decreased the K^+^ uptake of rice seedlings in the control group, and the NaCl treatment caused a more prominent effect on K^+^ uptake than the Na_2_CO_3_ treatment (Figure 7b). Bacterium-inoculated plants exhibited an opposite trend in ion uptake. Strain D2 could lower the Na^+^ accumulation in the rice seedlings under both normal and saline environments, and the most significant difference occurred in the NaCl treatment group (Figure 7a), while the K^+^ content was enhanced by D2 inoculation compared with the control group. Consequently, about 1.6-fold and 2.2-fold improvement in the K^+^/Na^+^ ratio was found by D2 inoculation in the NaCl and N_2_CO_3_ treatment groups, respectively (Figure 7c).

## 4. Discussion

As a growing issue worldwide, soil salinity causes detrimental impacts on agricultural production. Rice is a major cereal crop and has suffered yield losses due to soil salinization [2]. The application of PGPR is an effective and eco-friendly strategy for improving rice growth and yield under saline conditions [11]. In the present study, we isolated and characterized a halotolerant bacterium (isolate D2) from rice which showed PGP potential for rice exposed to both neutral and alkaline salts. Strain D2 was identified as *Enterobacter asburiae* based on phylogenetic analysis and genome sequencing. Although several salt-tolerant *Enterobacter* strains with PGP traits have been isolated from the rhizosphere of rice, their application in rice growth promotion was generally tested by the commonly used neutral salt NaCl [9,29,30]. Limited research has focused on exploring salt-tolerant *Enterobacter* species that can ameliorate salt stress in rice using alkaline salt. Therefore, we further characterized some common PGP traits and related genes of D2 and analyzed the inoculation effect on rice growth under neutral and alkaline salt stress.

Genome sequence analysis revealed that strain D2 contained multiple PGP-related genes and some common PGP traits were validated by qualitative analysis. Production of plant hormone IAA by concerting tryptophan in root exudates is a common strategy used by PGPR to enhance plant growth. The exogenous IAA synthesized by PGPR helps root growth and development, which facilitates the nutrient and water uptake of plants [8]. Habibi et al. isolated 98 PGPR from different rice cultivars and found that 89.7% of them produced IAA [31]. Although the IAA yield ranged from 2.0 to 92.4 mg/L, most of these PGPR produced IAA with a concentration below 20 mg/L [31], which was comparable to that of *E. asburiae* D2. Genome analysis showed that D2 had the tryptophan biosynthesis gene cluster (*trpA/B/CF/E/GD/S*), indicating its ability to produce IAA with either exogenous or endogenous tryptophan as a precursor [32]. In addition, the presence of the crucial indole-3-pyruvate decarboxylase gene (*ipdC*) in D2 suggests that this bacterium synthesizes IAA through the indole-3-pyruvic acid (IPA) pathway. The first step of this pathway is the conversion of tryptophan to IPA, which can be catalyzed by many amino acid aminotransferases (AATs). Then, IPA is converted by indole-3-pyruvate decarboxylase to form the intermediate indole-3-acetaldehyde (IAAld), which is further oxidized into IAA by an aldehyde dehydrogenase [33]. Except for *ipdC*, we identified an aspartate aminotransferase gene (*aspC*) in *E. asburiae* D2, which has been reported to be involved in the first step of the IPA pathway [34]. Meanwhile, two aldehyde dehydrogenase genes (*aldB* and *ALDH*) that might be responsible for the last step in the IPA pathway were found in strain D2 [33].

ACC deaminase production is another strategy used by PGPR to stimulate plant growth, especially under stressful conditions [8]. As a stress signal, the phytohormone ethylene regulates plant response to environmental stresses. However, the excessive accumulation of stress-induced ethylene causes an inhibitory effect on plant growth [35]. PGPR with ACC deaminase activities can lower the ethylene level of plants by degrading its precursor ACC in the root exudates [8,35]. While strain D2 showed a low ACC deaminase activity, it lacks the gene coding for ACC deaminase (*acdS*). Instead, a D-cysteine desulfhydrase gene (*dcyD*) was found in the genome of D2. As a homolog of ACC deaminase, D-cysteine desulfhydrase has been reported to be responsible for the ACC deaminase activity of *E. cloacae* Rs-2 without the *acdS* gene [36].

Despite phosphorus being one of the essential plant nutrients, it is usually present in relatively insoluble forms, which limits its availability [37]. *E. asburiae* D2 exhibited a high ability to solubilize inorganic phosphate, which could enhance phosphorus availability to plants and benefit their growth. This process is mainly associated with the organic acids secreted by PGPR [37], which was confirmed by the accompanied decrease in medium pH during the incubation process of D2. Gluconic acid is the most frequently reported organic acid for phosphate solubilization by PGPR, which is produced by the direct oxidation of glucose through glucose dehydrogenase (GDH) [37]. We found the GDH gene (*gcd*) in the genome of D2, proving its capacity to produce gluconic acid. In addition, two phosphatase genes (*phoA* and *aphA*) and a series of *phn* genes were also identified in D2, suggesting its ability in mineralizing organic phosphorus. Siderophores produced by PGPR play an important role in improving iron availability for plant uptake. These small compounds are metal chelators showing a high affinity for iron, and their complex can be easily accessed by plants [38]. The siderophore-producing *E. asburiae* D2 contains the *ent* and *iuc* gene clusters for the biosynthesis of enterobactin and aerobactin, which are catecholate and hydroxamate siderophores, respectively [38]. Similarly, some *Enterobacter* species like *E. cloacae* [39] and *E. asburiae* [40] have been reported to carry functional genes to produce these two siderophores. Furthermore, the presence of some nitrogen fixation-related genes was confirmed in the genome of *E. asburiae* D2. The above results confirmed that this bacterium has the ability to improve nutrient uptake and regulate the hormone level of plants, which is beneficial for plant growth under stressful environments.

Besides the above-mentioned genes related to PGP traits, the genome of strain D2 contains various genes that might be involved in improving salinity tolerance in plants. The phytohormone cytokinin contributes to plant growth, development, and stress alleviation. The existence of tRNA recycling genes (*miaA/B/E*) involved in cytokinin synthesis implies that strain D2 is able to produce cytokinin, which might play a role in regulating plant salt tolerance [41]. Accumulation of osmolytes plays an important role in plant tolerance to salinity. In the genome of strain D2, we identified genes related to synthesizing proline, glycine–betaine, and trehalose (Table S4). Plants could uptake these osmolytes produced by PGPR, which may act in synergism with endogenous osmolytes to improve plant salinity tolerance [8]. Moreover, strain D2 contains genes associated with exopolysaccharides production. Exopolysaccharides not only facilitate microbial colonization of plant roots but also protect plants from salt stress. They can bind with cations like Na^+^, and thus reduce the ionic toxicity in plants by restricting Na^+^ influx [8].

Salinity adversely impacts plant growth by inducing physiological and biochemical changes in plants such as reduction of photosynthesis, imbalance of ion homeostasis, and accumulation of ROS [42]. Since *E. asburiae* D2 could efficiently promote rice growth under salt stress, we investigated the role of D2 in alleviating salt stress in rice by evaluating some biochemical parameters. High salt levels reduce the content of chlorophyll by limiting its biosynthesis as well as inducing enzymatic degradation [42]. Both NaCl and Na_2_CO_3_ treatment resulted in a reduction in chlorophyll content in the rice leaves (Figure 5a). However, the amount of chlorophyll was significantly increased by strain D2 inoculation. The restoration of chlorophyll showed an improvement in photosynthetic efficiency of rice seedlings induced by strain D2. Proline is a common osmolyte that contributes to stabilizing cell structure, maintaining cellular redox potential, and scavenging ROS [42]. The accumulation of proline in rice seedlings was further enhanced by strain D2 in the presence of NaCl or Na_2_CO_3_ (Figure 5c), which was beneficial for rice to overcome the salt-induced osmotic shock and oxidative damage. Similarly, an increase in proline accumulation was observed in rice inoculated with *Bacillus pumilus* strain JPVS11 under salt stress [10]. PGPR-mediated proline accumulation in plants can be achieved through upregulating the expression of proline biosynthesis-related genes or absorbing exogenous proline that is provided by PGPR [8].

ROS production induced by salt stress causes a series of damages in plant cells [42]. MDA is an indicator of lipid peroxidation [30]. The MDA content significantly increased in rice exposed to salt (Figure 5d), indicating an accumulation of ROS induced by salt stress. Alleviation of salt-induced oxidative damage was observed by the decrease of MDA content in D2-inoculated rice (Figure 5d). An important strategy for plants to combat ROS is their enzymatic antioxidant systems such as SOD, POD, and CAT [42]. Inoculation of D2 led to a reduction in the activities of these ROS-scavenging enzymes, which also suggested an amelioration of oxidative stress in the rice seedlings under saline circumstances (Figure 6). Moreover, strain D2 was able to maintain ion homeostasis in salt-stressed rice seedlings, which was achieved by lowering Na^+^ accumulation and increasing K^+^ uptake (Figure 7). Consequently, the ionic toxicity induced by excessive Na^+^ was reduced.

## 5. Conclusions

We isolated and identified a halotolerant *Enterobacter asburiae* D2 from the rhizosphere of rice. It possesses multiple PGP properties including IAA and siderophore production, phosphate solubilization, and ACC deaminase activity. Genome analysis proved the existence of functional genes involved in plant growth promotion. *E. asburiae* D2 could confer resistance against both neutral and alkaline salts in rice by increasing chlorophyll and proline content, reducing oxidant damage, and maintaining ion homeostasis. Therefore, it could be applied as an efficient PGPR for tackling salinity in sustainable agriculture. Future work needs to evaluate the productivity of rice inoculated with strain D2 under saline conditions.

## Figures and Tables

**Table 1:** Physiological and biochemical characteristics of *Enterobacter* sp. D2.

Characteristics	Results	Characteristics	Results
Oxidase	−	Hydrolysis of:	
Phenylalanine deaminase	−	Gelatin	−
Lysine decarboxylase	+	Starch	−
β-galactosidase	+	Acid production from:	
Arginine dihydrolase	+	Glucose	+
Urease	−	Lactose	−
Nitrate reduction	+	Fructose	+
H_2_S production	−	Mannitol	+
Indole production	−	Inositol	−
Voges-Proskauer test	+	Sorbitol	+
Methyl red test	−	Rhamnose	−
Utilization of:		Sucrose	−
Malonate	+	Arabinose	−
Citrate	+	Melibiose	+

Note: +, positive result; −, negative result.

**Table 2:** Effect of bacterial inoculation on growth parameters of rice exposed to saline conditions.

Treatment		Root Length (cm)	Shoot Length (cm)	Root Fresh Weight (mg)	Shoot Fresh Weight (mg)	Root Dry Weight (mg)	Shoot Dry Weight (mg)
Water	Control	3.2 ± 0.3 b	23.7 ± 2.0 b	2.8 ± 0.3 c	104.8 ± 6.1 b	1.4 ± 0.3 b	20.8 ± 3.8 b
	D2	4.7 ± 0.3 a	28.0 ± 2.4 a	4.2 ± 0.4 a	125.3 ± 8.2 a	2.2 ± 0.1 a	24.4 ± 2.3 a
NaCl	Control	2.1 ± 0.5 c	18.2 ± 3.4 d	1.1 ± 0.3 d	55.3 ± 7.7 d	0.8 ± 0.1 c	13.1 ± 2.0 d
	D2	3.3 ± 0.6 b	21.8 ± 1.0 bc	2.8 ± 0.3 c	86.5 ± 7.2 c	1.4 ± 0.1 b	19.7 ± 1.3 bc
Na_2_CO_3_	Control	2.7 ± 0.5 bc	19.9 ± 2.7 cd	2.5 ± 0.3 c	92.8 ± 1.9 c	0.8 ± 0.2 c	16.7 ± 3.0 c
	D2	3.4 ± 0.5 b	26.8 ± 1.7 a	3.7 ± 0.1 b	103.3 ± 5.1 b	2.0 ± 0.2 a	21.8 ± 2.7 ab

Note: Different letters indicate a significant difference (*p* < 0.05).

## Data Availability

The 16S rRNA gene sequence of *Enterobacter* sp. D2 has been deposited in the GenBank database with the accession number MN540931. The GenBank accession number for the genome sequence of *Enterobacter* sp. D2 is JAXQPP000000000.

## Supplementary Materials

The following supporting information can be downloaded at: https://www.mdpi.com/article/10.3390/jox14010021/s1, Table S1: Plant growth-promoting abilities of bacteria isolated from the rhizosphere of rice; Table S2: Genomic features of *Enterobacter asburiae* D2; Table S3: Average nucleotide identity (ANI) (%) of *Enterobacter asburiae* D2 and other closely related *Enterobacter* species based on genome alignments; Table S4: Genes related to PGP traits and stress response in the genome of *Enterobacter asburiae* D2; Figure S1: Clusters of Orthologous Groups (COGs) function classification of *Enterobacter asburiae* D2 genome; Figure S2: Assignment of Gene Ontology (GO) term for predicted genes of *Enterobacter asburiae* D2 genome; Figure S3: Kyoto Encyclopedia of Genes and Genomes (KEGG) annotation of *Enterobacter asburiae* D2 genome.
