# Supplementary material for: Mitigation of Salt Stress in Rice by the Halotolerant Plant Growth-Promoting Bacterium Enterobacter asburiae D2
Source: J Xenobiot. 2024 Mar 1;14(1):333–49. doi: 10.3390/jox14010021 (PMC10971743; doi:10.3390/jox14010021)
Supplement: Supplementary file 1 [file jox-14-00021-s001.zip › jox-2827387-supplementary.pdf]

Mitigation of Salt Stress in Rice by the Halotolerant Plant Growth-Promoting Bacterium *Enterobacter asburiae* D2

Zican Ning [1,2], Kexin Lin [1], Mengya Gao [1], Xiao Han [1], Qingjie Guan [1,2], Xiang Ji [3,4], Shuyu Yu [3,*] and Lei Lu [1,2,*]

[1] College of Life Sciences, Northeast Forestry University, Harbin 150040, China; zicanning@163.com (Z.N.); kx_lin@126.com (K.L.); mengyagao@yeah.net (M.G.); xiaohan093@163.com (X.H.); qingjieguan@126.com (Q.G.)
[2] Key Laboratory of Saline-Alkali Vegetation Ecology Restoration (Northeast Forestry University), Ministry of Education, Harbin 150040, China
[3] College of Water Conservancy and Civil Engineering, Inner Mongolia Agricultural University, Hohhot 010018, China; jixiang@imau.edu.cn
[4] Hetao College, Bayan Nur 015000, China
* Correspondence: shuyuyu@126.com (S.Y.); llu@nefu.edu.cn (L.L.)

Table S1. Plant growth-promoting abilities of bacteria isolated from the rhizosphere of rice.

Strains	Phosphate solubilization	Siderophore production	ACC deaminase activity
D2	++	+	+
D5	++	+	−
D6	−	+	+
D8	−	−	−
D11	+	++	−
D15	−	−	+

Note: −, not detected; +, low activity; ++, strong activity.

Table S2. Genomic features of *Enterobacter asburiae* D2.

Features	Values
Genome size (bp)	4,659,687
G+C content (%)	55.73
Protein-coding genes (CDS)	4327
Gene total length (bp)	4,158,297
Average gene length (bp)	961.01
Gene/Genome (%)	89.24
rRNAs (5s; 16s; 23s)	8; 1; 0
tRNAs	74
NR annotation	4,316
Swiss-Prot annotation	3,896
Pfam annotation	4,021
COG annotation	3,779
GO annotation	3,160
KEGG annotation	3,082

Table S3. Average nucleotide identity (ANI) (%) of *Enterobacter asburiae* D2 and other closely related *Enterobacter* species based on genome alignments.

Strain	ANI value (%)
Enterobacter sp. D2	
Enterobacter mori LMG 25706(T)	90.05
Enterobacter asburiae CAV1043	98.29
Enterobacter cloacae ATCC 23373(T)	88.78
Enterobacter hormaechei ATCC 49162(T)	87.65
Enterobacter ludwigii EN-119(T)	88.39
Enterobacter roggenkampii EN-117(T)	93.11
Enterobacter cancerogenus ATCC33241(T)	86.79
Enterobacter sichuanensis WCHECl1597(T)	91.57
Enterobacter kobei DSM 13645(T)	90.65
Enterobacter bugandensis EB-247(T)	91.47

Table S4. Genes related to PGP traits and stress response in the genome of *Enterobacter asburiae* D2.

PGP traits	Gene	Gene function
IAA production	*trpA*	Tryptophan synthase alpha chain [EC:4.2.1.20]
	trpB	Tryptophan synthase beta chain [EC:4.2.1.20]
	trpCF	Indole-3-glycerol phosphate synthase [EC:4.1.1.48]
	trpE	Anthranilate synthase component I [EC:4.1.3.27]
	trpGD	Anthranilate synthase [EC:4.1.3.27]
	trpS	Tryptophanyl-tRNA synthetase [EC:6.1.1.2]
	mtr	Tryptophan permease
	aspC	Aspartate aminotransferase [EC:2.6.1.1]
	ipdC	Indole-3-pyruvate decarboxylase [EC:4.1.1.74]
	aldB	Aldehyde dehydrogenase [EC:1.2.1.-]
	ALDH	Aldehyde dehydrogenase (NAD+) [EC:1.2.1.3]
Phosphate solubilization	*pstA/C*	Phosphate ABC transporter permease
	pstB	Phosphate ABC transporter ATP-binding protein [EC:7.3.2.1]
	pstS	Phosphate ABC transporter substrate-binding protein
	phoU	Phosphate transport system protein
	phnC	Phosphonate ABC transporter ATP-binding protein [EC:7.3.2.2]
	phnD	Phosphonate ABC transporter substrate-binding protein
	phnE	Phosphonate transport system permease protein
	phnF	Phosphonate metabolism transcriptional regulator
	phnG/H/I/L	Alpha-D-ribose 1-methylphosphonate 5-triphosphate synthase subunit [EC:2.7.8.37]
	phnJ	Alpha-D-ribose 1-methylphosphonate 5-phosphate C-P lyase [EC:4.7.1.1]
	phnK	Putative phosphonate transport system ATP-binding protein
	phnM	Alpha-D-ribose 1-methylphosphonate 5-triphosphate diphosphatase [EC:3.6.1.63]
	phnN	Ribose 1,5-bisphosphokinase [EC:2.7.4.23]
	phnO	Aminoalkylphosphonate N-acetyltransferase [EC:2.3.1.280]
	phnP	Phosphoribosyl 1,2-cyclic phosphate phosphodiesterase [EC:3.1.4.55]
	gcd	Quinoprotein glucose dehydrogenase [EC:1.1.5.2]
	phoA	Alkaline phosphatase [EC:3.1.3.1]
	aphA	Acid phosphatase (class B) [EC:3.1.3.2]

Table S4. *Cont.*

PGP traits	Gene	Gene function
Nitrogen fixation	*nifJ*	Pyruvate-ferredoxin oxidoreductase [EC:1.2.7.1]
	iscA	iron-sulfur cluster assembly protein
	iscR	Iron-sulfur cluster assembly transcription factor
	iscS	Cysteine desulfurase [EC:2.8.1.7]
	iscU	Nitrogen fixation protein NifU and related proteins
	sufA/B/D	Fe-S cluster assembly protein
	sufC	Fe-S cluster assembly ATP-binding protein
	sufE	cysteine desulfuration protein
	sufS	cysteine desulfurase
ACC deaminase activity	*dcyD*	D-cysteine desulfhydrase [EC:4.4.1.15]
Siderophore production	*fepA*	Ferric enterobactin receptor
	fepB	Ferric enterobactin transport system substrate-binding protein
	fepC	Iron-siderophore transport system ATP-binding protein
	fepD/G	Iron-siderophore transport system permease protein
	afuA	Iron (III) transport system substrate-binding protein
	afuB	Iron (III) transport system permease protein
	afuC	Iron (III) transport system ATP-binding protein [EC:7.2.2.7]
	fhuB	Ferric hydroxamate transport system permease protein
	fhuC	Ferric hydroxamate transport system ATP-binding protein [EC:7.2.2.16]
	fhuD	Ferric hydroxamate transport system substrate-binding protein
	efeB	Iron uptake transporter deferrochelatase [EC:1.11.1.-]
	efeO	Iron uptake system component
	efeU	High-affinity iron transporter
	entA	2,3-dihydro-2,3-dihydroxybenzoate dehydrogenase [EC:1.3.1.28]
	entB	Bifunctional isochorismate lyase [EC:3.3.2.1]
	entC	Isochorismate synthase [EC:5.4.4.2]
	entD	Enterobactin synthetase component D [EC:6.3.2.14]
	entE	(2,3-dihydroxybenzoyl)adenylate synthase [EC: 6.2.1.71]
	entF	Enterobactin non-ribosomal peptide synthetase [EC: 6.2.1.72]
	entH	Proofreading thioesterase
	entS	Enterobactin transporter
	iucA	N2-citryl-N6-acetyl-N6-hydroxylysine synthase [EC:6.3.2.38]
	iucB	Acetyl CoA:N6-hydroxylysine acetyl transferase [EC:2.3.1.102]
	iucC	Aerobactin synthase [EC:6.3.2.39]
	iucD	Lysine N6-hydroxylase [EC:1.14.13.59]
Cytokinin synthesis	*miaA*	tRNA dimethylallyltransferase [EC:2.5.1.75]
	miaB	tRNA-2-methylthio-N6-dimethylallyladenosine synthase [EC:2.8.4.3]
	miaE	tRNA-2-(methylsulfanyl)-N6-isopentenyladenosine37 hydroxylase [EC:1.14.99.69])
Glycine-betaine synthesis	*betA*	Choline dehydrogenase [EC:1.1.99.1]
	betB	Betaine-aldehyde dehydrogenase [EC:1.2.1.8]
	betT	Choline/glycine/proline betaine transport protein

Table S4. *Cont.*

PGP traits	Gene	Gene function
Proline synthesis	*proA*	Glutamate-5-semialdehyde dehydrogenase [EC:1.2.1.41]
	proB	Glutamate 5-kinase [EC:2.7.2.11])
	proC	Pyrroline-5-carboxylate reductase [EC:1.5.1.2]
	proP	MFS transporter, MHS family, proline/betaine transporter
	proS	Prolyl-tRNA synthetase [EC:6.1.1.15]
	proV	Glycine betaine/proline transport system ATP-binding protein [EC:7.6.2.9]
	proW	Glycine betaine/proline transport system permease protein
	proX	Glycine betaine/proline transport system substrate-binding protein
	proY	Proline-specific permease ProY
Trehalose synthesis	*treB*	Trehalose PTS system EIIBC or EIIBCA component [EC:2.7.1.201]
	treS	Maltose alpha-D-glucosyltransferase [EC:5.4.99.16]
	treY	(1->4)-alpha-D-glucan 1-alpha-D-glucosylmutase [EC:5.4.99.15]
	treZ	Maltooligosyltrehalose trehalohydrolase [EC:3.2.1.141]
	otsA	Tehalose 6-phosphate synthase [EC:2.4.1.15 2.4.1.347]
	otsB	Trehalose 6-phosphate phosphatase [EC:3.1.3.12]
EPS synthesis	*bcsA*	Cellulose synthase (UDP-forming) [EC:2.4.1.12]
	bcsB/C	Cellulose synthase operon protein B/C
	bcsZ	Endoglucanase [EC:3.2.1.4]
	wcaA/B/C/E/F/I/L	Colanic acid biosynthesis glycosyltransferase
	wcaD	Colanic acid polymerase
	wcaJ	Undecaprenyl-phosphate glucose phosphotransferase [EC:2.7.8.31]
	wcaK	Colanic acid biosynthesis pyruvyl transferase
	wcaM	Colanic acid biosynthesis protein WcaM
	pgaA	Poly-beta-1,6 N-acetyl-D-glucosamine export porin PgaA
	pgaB	Poly-beta-1,6-N-acetyl-D-glucosamine N-deacetylase
	pgaC	Poly-beta-1,6-N-acetyl-D-glucosamine synthase
	pgaD	Poly-beta-1,6-N-acetyl-D-glucosamine biosynthesis protein

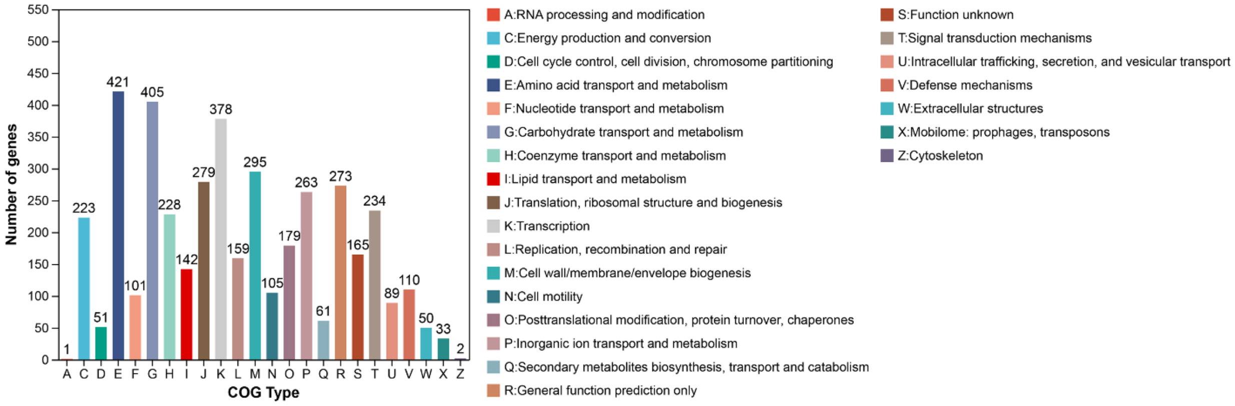

Figure S1. Clusters of Orthologous Groups (COGs) function classification of *Enterobacter asburiae* D2 genome.

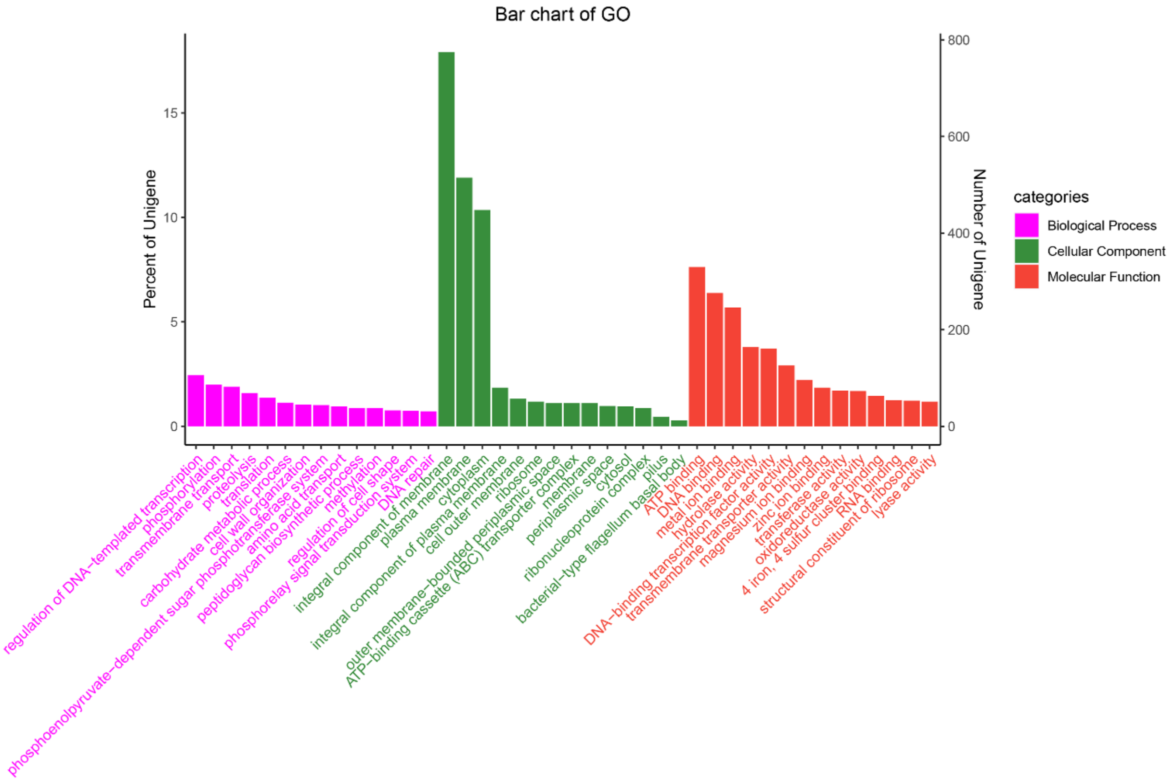

Figure S2. Assignment of Gene Ontology (GO) term for predicted genes of *Enterobacter asburiae* D2 genome.

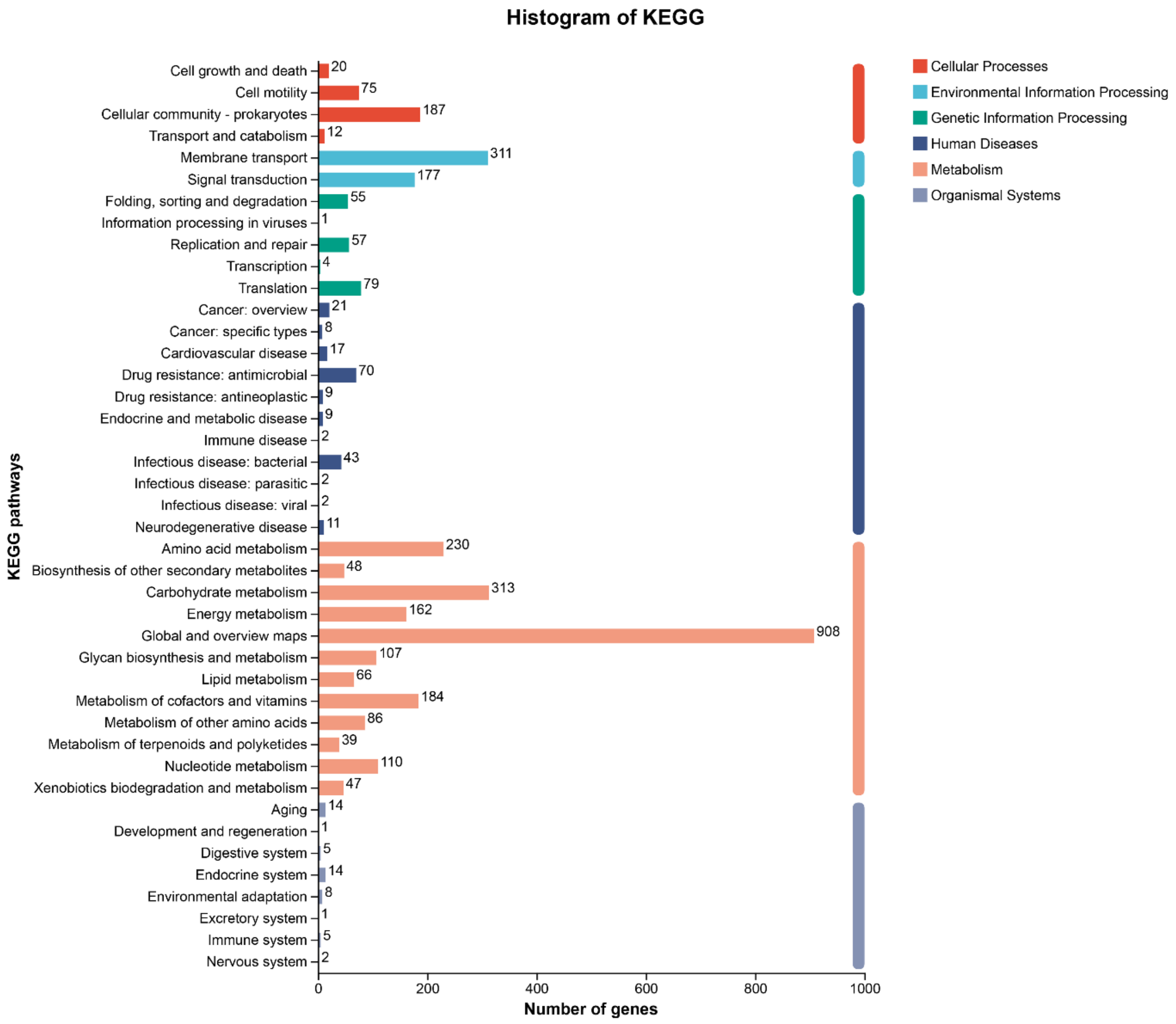

Figure S3. Kyoto Encyclopedia of Genes and Genomes (KEGG) annotation of *Enterobacter asburiae* D2 genome.
